# Density propagation based adaptive multi-density clustering algorithm

**DOI:** 10.1371/journal.pone.0198948

**Published:** 2018-07-18

**Authors:** Yizhang Wang, Wei Pang, You Zhou

**Affiliations:** 1 College of Computer Science and Technology, Jilin University, Changchun, China; 2 Key Laboratory of Symbolic Computation and Knowledge Engineering of Ministry of Education, Changchun, China; 3 Department of Computing Science, University of Aberdeen, United Kingdom; Northeast Normal University, CHINA

## Abstract

The performance of density based clustering algorithms may be greatly influenced by the chosen parameter values, and achieving optimal or near optimal results very much depends on empirical knowledge obtained from previous experiments. To address this limitation, we propose a novel density based clustering algorithm called the Density Propagation based Adaptive Multi-density clustering (DPAM) algorithm. DPAM can adaptively cluster spatial data. In order to avoid manual intervention when choosing parameters of density clustering and still achieve high performance, DPAM performs clustering in three stages: (1) generate the micro-clusters graph, (2) density propagation with redefinition of between-class margin and intra-class cohesion, and (3) calculate regional density. Experimental results demonstrated that DPAM could achieve better performance than several state-of-the-art density clustering algorithms in most of the tested cases, the ability of no parameters needing to be adjusted enables the proposed algorithm to achieve promising performance.

## Introduction

Clustering has been a promising technique in data mining and pattern recognition [1–3]. One important goal of clustering is to find potential data structures without supervision information. In density based clustering algorithms, a density threshold (which often represents radius of searching circle and size of grids) is usually used to build a search framework, and the final results depend on the local or global density threshold settings. Density based clustering algorithm follows the hypothesis that the high-density regions are always surrounded by low-density connected sets. DBSCAN, OPTICS, DENCLUE, CLIQUE all belong to this kind of algorithms [4–7], which use thresholds to find clusters. In addition to the above algorithms, recently Rodriguez and Laio proposed a novel fast density clustering algorithm by searching density peaks (DP) [8], and they adopted a global threshold to calculate the local density of each point. However, manual intervention and domain knowledge is still required to obtain an appropriate threshold in DP, and a user may require a significant amount of time to learn how to configure parameters properly [9]. Based on the above consideration, in this research, we propose an adaptive density clustering method to address the above issue.

To determine appropriate parameter values for clustering algorithms, a number of approaches have been used, for instance, swarm-inspired algorithms and genetic algorithms [10–13]. The swarm algorithms can be used to find the optimal parameters, and examples of swarm algorithms include particle swarm optimization (PSO) and fireworks algorithm [14, 15]. However, the parameter search process needs prior knowledge to calculate fitness functions in these algorithms, and it is challenging to design proper fitness functions. For unsupervised clustering tasks without ground-truth labels, the swarm optimization algorithms and genetic algorithms are not straightforward to perform parameter search. In addition, the chosen parameter values may be invalid when clustering new datasets with different characteristics. This necessitates an adaptive approach which can automatically achieve density clustering. Thus, in this research we will develop a robust and fast adaptive clustering strategy from a new perspective of density propagation.

The significant contributions of this research are twofold. First, the definitions of between-class margin and within-class cohesion of multi-density structure are proposed for achieving density propagation. Second, the DPAM algorithm is proposed to automatically extract potential spatial data structure without manual adjustment of parameter values.

The rest of this paper is organized as follows: in Section 2, related work about density clustering is reviewed. In Section 3, the proposed DPAM algorithm is described in details. Experimental results and associated analysis on publicly available datasets commonly used for evaluating clustering algorithms are reported in Section 4. The paper ends with conclusions in Section 5.

## Related work

In this section, we review the related work of density clustering algorithms. Most density based clustering algorithms are based on the same assumption: the dense regions of objects surrounded by low-density regions clusters. Based on this assumption, many methods such as DBSCAN, OPTICS, DENCLUE, CLIQUE and STING are proposed [16]. These algorithms calculate local density according to a given distance metric, which contains the minimum number of neighborhood at least. DBSCAN is a typical density based clustering algorithm, and in DBSCAN the density of every point is associated with the number of points within a threshold radius circle. OPTICS aims to overcome the shortcomings of DBSCAN by ordering points to identify the cluster structure. The data space can be quantized into a finite number of cells to form a grid structure. There also exist some grid density clustering algorithms: STING uses grids to store statistical information by the wavelet transform method; CLIQUE employs grids to high dimensional data clustering [17, 18]. However, all these algorithms require users to set parameter values manually and this may lead to fluctuating clustering performance.

DP is an outstanding density clustering algorithm [8], and it adjusts the density hypothesis: it assumes that the low density areas cover centers of clusters, and the centers are far away from each other. In the DP algorithm, an important parameter *d*_*c*_ (the percent of all data points similarities) representing the global density should be specified by users empirically, and it is a critical parameter which fundamentally affects the algorithm performance and the cluster centers produced by decision graph [19]. However, it may be very challenging to obtain satisfactory results in DP without prior knowledge.

In general, all of the above algorithms are dependent on the choice of thresholds, such as the density size or grid size. To use these algorithms, users often need to adjust parameter values gradually according to situations specific to different datasets in order to achieve better clustering results.

## Density propagation based adaptive multi-density clustering (DPAM)

A single natural cluster can be composed of a set of micro-clusters, each of which includes a smaller number of points with a higher local density. We propose the concept of regional density to measure the distributions of points in each micro-cluster in Section 3.3. The proposed DPAM is based on the following assumption: the smaller the difference of regional density among different micro-cluster is, the better the clustering results are.

The points of different regional density constitute multi-density spatial data, and it is easier to cluster the micro-clusters than the original data. Hence a fast adaptive density clustering algorithm based on density propagation is proposed, which is better suited for clustering the obviously multi-density spatial data. DPAM mainly considers data of obvious boundaries except for links between clusters.

DPAM is a three-stage clustering algorithm: Stage 1 is establishing undirected graph (Section 3.1); Stage 2 is merging micro-clusters (Section 3.2); and Stage 3 is the final clustering (Section 3.3). DPAM does not depend on specific parameters to achieve accurate and robust results. We now describe these three stages in detail.

### Generate micro-clusters graph

The first stage of DPAM is to initially build sub-graphs of all micro-clusters to make sure that one micro-cluster only has one kind of label. There are several different methods for generating sub-graphs, such as *k* nearest neighbor graph and *ϵ*- neighbors (*ϵ* means the minimum number of data points that a sub-cluster contains) [20], but these methods cannot obtain pure labels within a single micro-cluster. We choose the affinity propagation (AP) clustering algorithm to guarantee that the points in the same micro-cluster has the same label [21]. AP tends to cluster local neighbor points to form micro-clusters [22]. Parameter preference (*P*) in the AP algorithm is an important value which can be varied to produce different clusters with different numbers of points [23]. The smaller the value of *P*, the bigger the final number of the clusters is [24], and the more likely every cluster will contain the same label. We use the characteristics of AP that easily distinguishes local dense points and dynamically divides the global structures to produce micro-clusters. We denote a data set of n points by *X* = {*X*_1_, …, *X*_*i*_, … *X*_*j*_, … *X*_*n*_}, and the similarity matrix *S*(*i*, *j*) is calculated by Euclidean distance as in Eq (1) [25], then we use Eq (2) to reset *P* resulting in a smaller number of clusters. In Eq (2) the default value of *θ* is 0.1 in order to produce more micro-clusters. We will show in Section 4.1 that the default value of *θ* is effective for all data sets tested in this research and it does not need to be modified, and the default value is just to obtain the pure label in a single micro-cluster as much as possible.

$$\begin{array}{c} S\left(i,j\right)=∥X_{i}-X_{j}∥ \end{array}$$ (1)

$$\begin{array}{c} P=\theta min(S(i,j)) \end{array}$$ (2)

Using the AP algorithm with the newly defined *P* that can effectively recognizes small local clusters, we are now able to obtain many micro-clusters. Every micro-cluster has only one kind of label, and these micro-clusters will constitute the final better clusters. Thus, the clustering task is transformed to grouping all these micro-clusters.

### Density propagation

DPAM uses the between-class margin (which measures distance between two micro-clusters) and within-class cohesion (which describes compactness within a single micro-cluster) measure to determine whether the two clusters can be merged into one. This idea is called density propagation. First all data are assigned to different *N* micro-clusters *C* = {*C*_1_, …, *C*_*k*_, … *C*_*l*_, … *C*_*N*_}. From the local density perspective, the closer two micro-clusters are, the more likely they may belong to the same class. Thus we use Eq (3) to represent the between-class margin (*BM*), which describes connectivity between two micro-clusters *C*_*k*_ and *C*_*l*_:

$$\begin{array}{c} BM=\min_{i\in C_{k},j\in C_{l},k\neq l}\left\{S\left(i,j\right)\right\} \end{array}$$ (3)

The within-class cohesion (*WC*) defined in Eq (4) describes the compactness of a micro-cluster. *WC* implies the maximum distance of any two points within a sub-cluster, and it measures the degree of closeness for a micro-clusters *C*_*k*_.

$$\begin{array}{c} WC=\max_{i\in C_{k},j\in C_{k}}\left\{S\left(i,j\right)\right\} \end{array}$$ (4)

Formula (5) is the decision function for deciding whether *C*_*k*_ and *C*_*l*_ can be combined into the same cluster. If the distance of one point of *C*_*k*_ and the next among *C*_*l*_ is smaller than *WC* of *C*_*k*_, we merge the two micro-clusters *C*_*k*_ and *C*_*l*_ into a common cluster, then we set *k* = *l*, which means all points in *C*_*k*_ will be labelled as *l*. In this way, all the labels of these micro-clusters will be modified by such density propagation mechanism.

$$\begin{array}{c} \left\{ \begin{array}{cc} k=l, & BM-WC<0\\ k\neq l, & others \end{array}\right. \end{array}$$ (5)

In this stage, the connective neighbor micro-clusters will be merged into the same clusters. The local decision method has its disadvantage: when one micro-cluster is comparatively far away from another, local density propagation will not work, and they cannot become a cluster. Thus we propose the concept of regional density to complete the final clustering from the global perspective. This will be described in Section 3.3.

### Calculate regional density

The high quality clusters are surrounded by neighbors with lower local density and they are at a relatively large distance and have higher local density [26]. Often, each sub-cluster has boundary points around it to form a region, so we propose the concept of regional density (*RD*) to detect micro-clusters which have high density, and then the sparse points are assigned to their nearest high density clusters. *RD* is influenced by two factors: the linear density of a single micro-cluster (*LD*) as defined in Eq (6) and the number of points within a single micro-cluster (*NS*). *RD* is the product of *LD* and the global proportion of *NS* (*NS*/*n*) as shown in Eqs (6) and (7):

$$\begin{array}{c} LD_{C_{k}}=\frac{\sum_{i=1,i+1\in C_{k},i+1\leq n,i\in C_{k}}^{n}S\left(i,i+1\right)}{NS} \end{array}$$ (6)

$$\begin{array}{c} RD_{C_{k}}=\frac{\sum_{i=1,i+1\in C_{k},i+1\leq n,i\in C_{k}}^{n}S\left(i,i+1\right)}{n} \end{array}$$ (7)

Essentially, density propagation is to identify locally compact clusters, and ultimately we would like to obtain results with strong between-class margin and within-class cohesion. A micro-cluster with a high *RD* value has higher density. The smaller the gaps of regional density among different micro-clusters are, the better the clustering results we can get. So we use the variance to measure regional density gaps of different sub-clusters and minimize the variance of *RD* among sub-clusters through density propagation. The process of minimizing the variance of *RD* is to achieve more robust clustering results, as shown in Formula (8).

$$\begin{array}{c} minVar(RD), \end{array}$$ (8)

where *RD* represents regional densities of all micro-cluster, *RD* = {*RD*_*C*_1__, ⋯, *RD*_*C*_*N*__}, *N* is the number of micro-clusters. We solve the above model by merging low density regions into their nearest higher density micro-clusters so as to update *RD*. We then calculate the variance of *RD* with the updated *RD* values. We repeat this process until the variance of *RD* cannot be further reduced. Eventually, results that fit our hypothesis will be obtained. The detailed steps of solving Formula (8) are shown in Algorithm 1.

**Algorithm 1: The Detailed Steps of Solving Formula (8)**

**repeat**

 1. Calculate *RD* for every micro-cluster;

 2. Calculate variance of all *RD*s

 3. Merge the micro-cluster *C*_*k*_ with low *RD* into their nearest high *RD* micro-cluster *C*_*l*_;

**until** Variance of all *RD*s no longer decreases

### The detailed procedure of DPAM

The DPAM algorithm begins with micro-clusters graphs produced by AP, which is a better generation method. Afterwards we calculate the distance of every two points, and we use between-class margin (*BM*) and within-class cohesion (*WC*) to evaluate which two micro-clusters can be merged into a new cluster. But there may still exist some sparse density regions, so we merge them into their nearest high density clusters according to the density assumptions. The detailed procedure of the proposed DPAM algorithm is shown in Algorithm 2.

**Algorithm 2: The Detailed Steps of the DPAM algorithm**

**Require**: Data set, Preference *P*.

**Ensure**: The clustered label results *Y* for *X*.

 1: Clustering by Affinity Propagation;

 2: **repeat**

 3:   Calculate *BM* and *WC* based on Eqs (3) and (4), respectively;

 4:   Propagate labels *Y* of micro-clusters according to the decision function given by Formula (5);

 5: **until** every label of micro-cluster is reset

 6: Solve Model (8) by Algorithm 1;

 7: Return *Y*

In conclusion, the time complexity of the DPAM is *O*(*n*^2^), which is not higher than DBSCAN and DP Clustering.

## Experimental results and discussion

In this section we report the performance of DPAM. We experimented with publicly available data, and the clustering performance evaluation tasks include: (1) whether DPAM can produce correct clustering results for low dimensional data, (2) performance comparison with other density clustering algorithms, and (3) whether DPAM is robust on high-dimensional data.

All the experiments are carried out with the same default parameter values and the same data. Eleven datasets are used in the experiments, as shown in Table 1, datasets Jain, Spiral and Flame are taken from http://cs.joensuu.fi/sipu/datasets/, dataset Dount is artificial, and it can be downloaded from https://github.com/mlyizhang/DPAM.git. Datasets breast, ionosphere, iris, sonar, vehicle, liver and wine can be downloaded from (http://archive.ics.uci.edu/ml/datasets.html).

**Table 1:** The features of datasets.

Datasets	Number of samples	Dimensions	Number of classes
*Jain*	373	2	2
*Spiral*	312	2	3
*Flame*	240	2	2
*Donut*	600	2	2
*breast*	277	9	2
*ionosphere*	351	34	2
*iris*	150	4	3
*sonar*	208	60	2
*vehicle*	846	18	7
*liver*	345	6	2
*wine*	178	13	3

To evaluate the clustering results, we adopt the commonly used evaluation index F-measure (*FM*), as defined below: for a pair of points *X*_*i*_ and *X*_*j*_, they are represented as *TP* if they have the same class and the same cluster. They are represented as *FP* if they have different class labels but are grouped into the same cluster. They are represented as *FN* if they have the same class label but are grouped into different clusters.

$$\begin{array}{c} precision=\frac{\#TP}{\#TP+\#FP} \end{array}$$ (9)

$$\begin{array}{c} Recall=\frac{\#TP}{\#TP+\#FN} \end{array}$$ (10)

$$\begin{array}{c} FM=\frac{2*Precision*Recall}{Precision+Recall} \end{array}$$ (11)

In Eqs (8) and (9), # represents the number of the corresponding quantity.

### Sensitivity analysis of micro-clusters generation

The first and important step of DPAM is generation of micro-clusters as described in Section 3.1. There is a parameter *θ* in this stage, and we give a default value *θ* = 0.1, but it never changes when new data sets are used.

**Fig 1:**
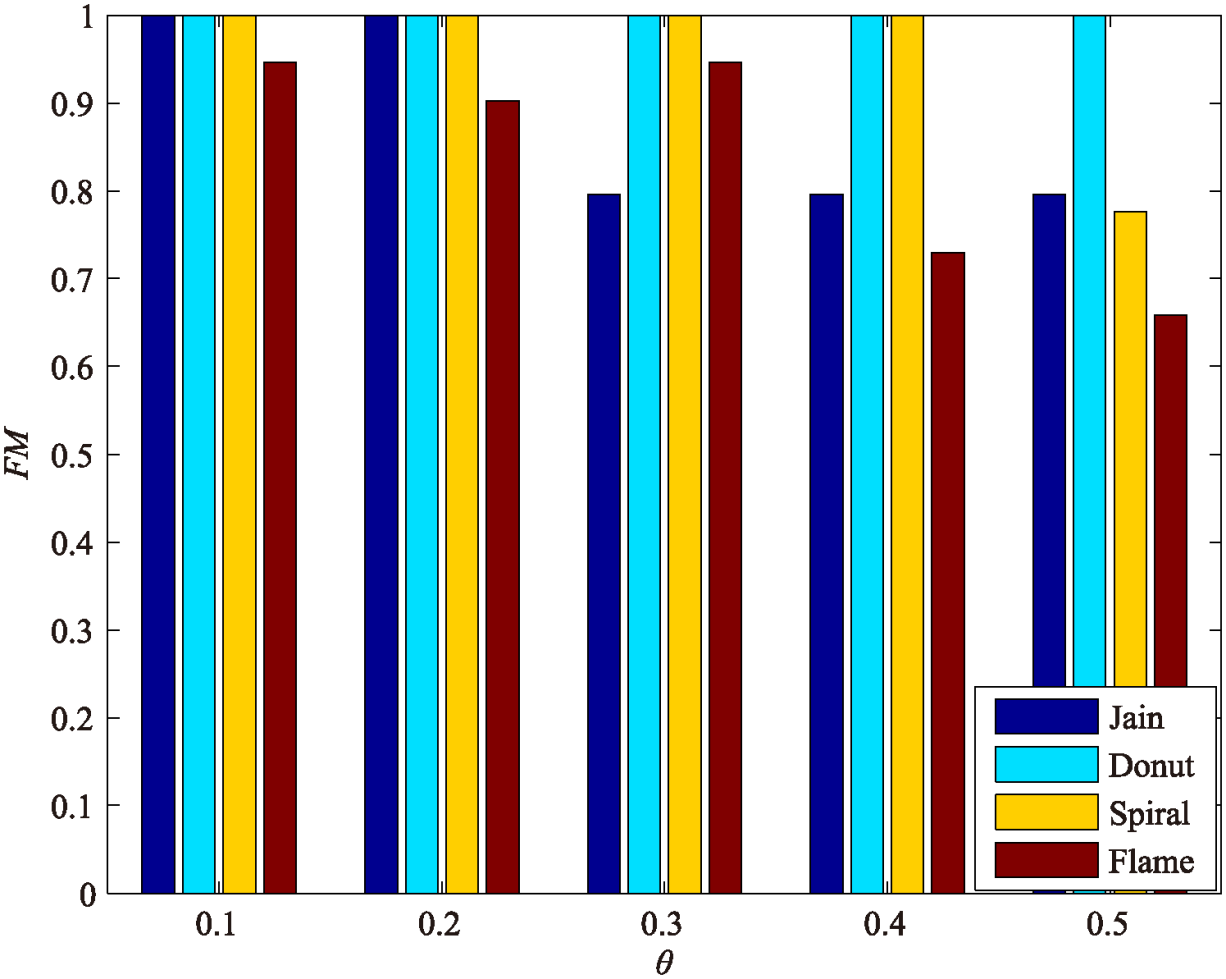
Influence of parameters on clustering results in four datasets.

To illustrate the effectiveness of this, we make an experiment on four datasets: one can see the influence of parameters *θ* on the clustering results for the four datasets in Fig 1: when *θ* becomes bigger, a single cluster produced by AP may more likely contain different labels, the performance of DPAM decreases. The default value of *θ* will ensure the generation of many high quality sub-clusters, and *θ* = 0.1 makes sure that we are more likely to obtain pure labels in a single micro-cluster when clustering different datasets. This unchanged parameter can work effectively for all the data sets tested in this work, so we hold this default value in all experiments to prove the effectiveness of the adaptive DPAM algorithm.

### The synthetic datasets

**Fig 2:**
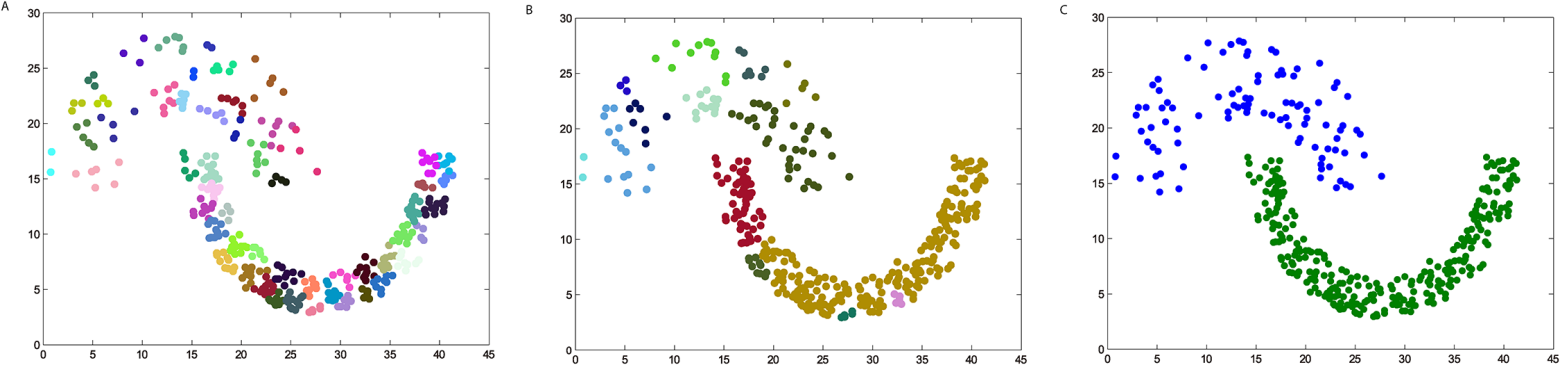
The three steps of DPAM on dataset Jain. (A) Stage 1, (B) Stage 2, (C) Stage 3.

We firstly verify the effectiveness of the DPAM algorithm on two dimensional datasets. The three steps of DPAM are shown in Table 2 and Figs 2∼5. DPAM does not need parameter adjustment and empirical knowledge, which is the greatest advantage of our method. But for the Flame dataset, in which there exist some links or relatively high-density points between clusters, the performance of our method is not as good as that of other datasets. For the Spiral and Donut datasets, the points are relatively evenly distributed, so good performance can be achieved even in the first two stages.

**Table 2:** The three steps of DPAM on synthetic datasets.

Datasets	*FM* of Stage 1	*FM* of Stage 2	*FM* of Stage 3
*Jain*	0.2164	0.9831	1
*Spiral*	0.2369	1	1
*Flame*	0.2226	0.8117	0.9463
*Donut*	0.1749	1	1

**Fig 3:**
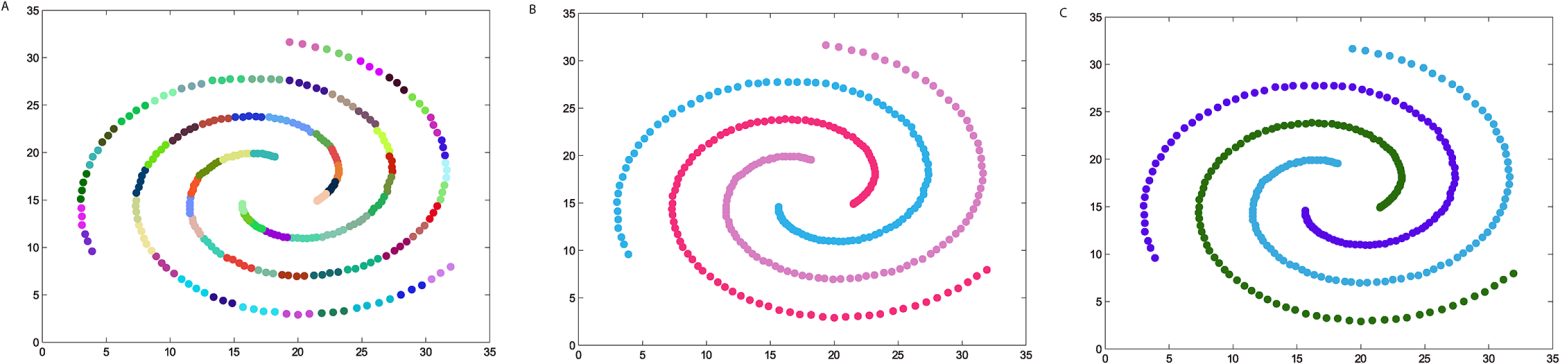
The three steps of DPAM on dataset Spiral. (A) Stage 1, (B) Stage 2, (C) Stage 3.

**Fig 4:**
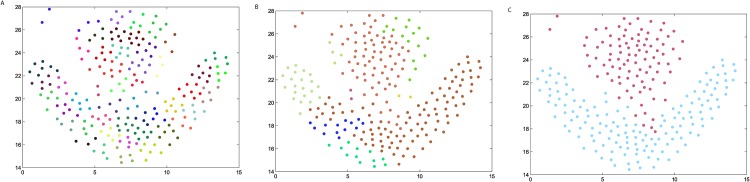
The three steps of DPAM on dataset Flame. (A) Stage 1, (B) Stage 2, (C) Stage 3.

**Fig 5:**
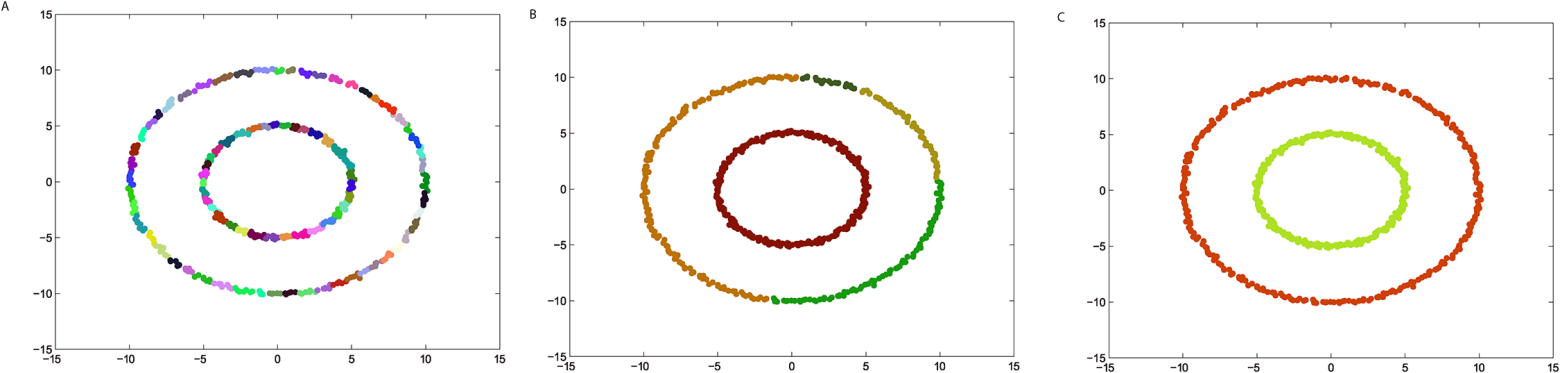
The three steps of DPAM on dataset Donut. (A) Stage 1, (B) Stage 2, (C) Stage 3.

**Fig 6:**
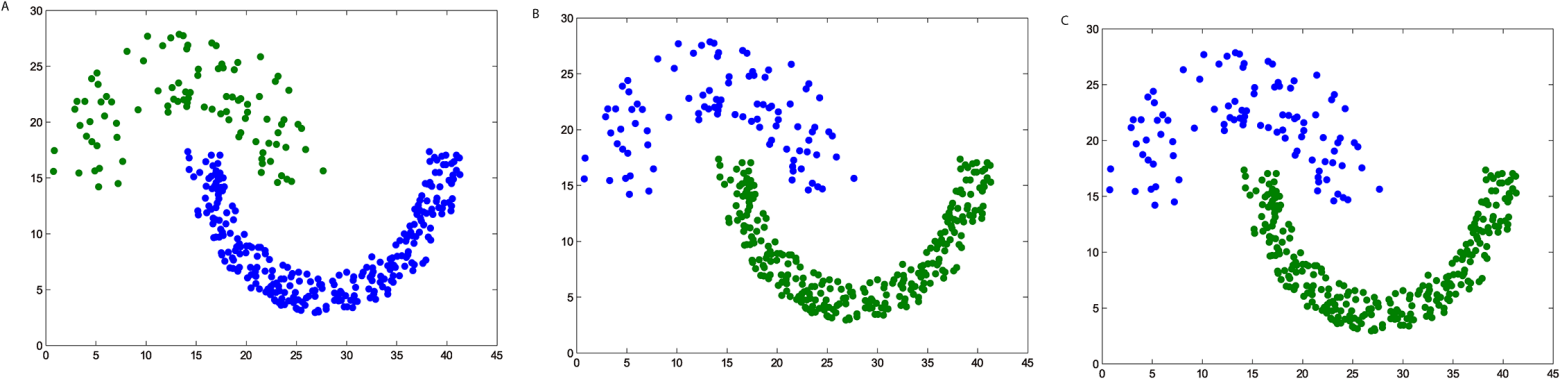
The clustering results of DBSCAN, DP Clustering and DPAM using Jain dataset. (A) DBSCAN (*MinPts* = 2.9, *Eps* = 20), (B) DP (*percent* = 40), (C) DPAM.

To further demonstrate the effectiveness of our algorithm, we compare it with other density clustering algorithms and report the results in Figs 6∼9 and Table 3. The parameters of DBSCAN and DP are turned through trial and error to obtain the best results. DBSCAN has two parameters: *MinPts* and *Eps*, and different results are produced by different values. DP Clustering has just one parameter *d*_*c*_, but its decision phase of cluster centers still needs to be chosen, and sometimes it is difficult to choose how many center points (number of center represents cluster number).

**Fig 7:**
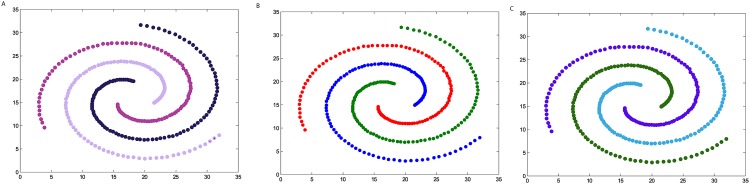
The clustering results of DBSCAN, DP Clustering and DPAM using Spiral dataset. (A) DBSCAN (*MinPts* = 2.5, *Eps* = 2), (B) DP (*percent* = 5), (C) DPAM.

**Fig 8:**
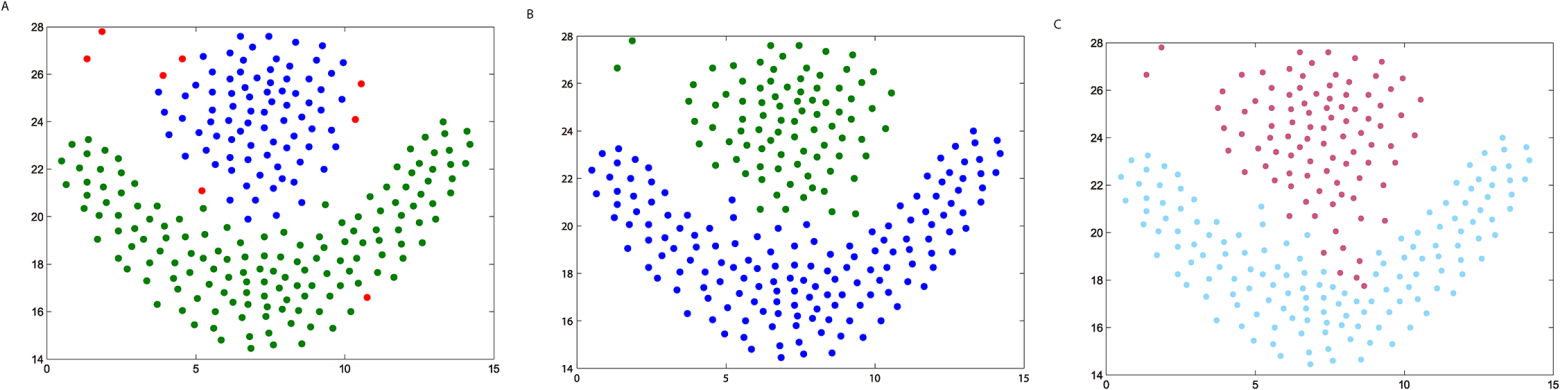
The clustering results of DBSCAN, DP Clustering and DPAM using Flame dataset. (A) DBSCAN (*MinPts* = 1, *Eps* = 6), (B) DP (*percent* = 5), (C) DPAM.

**Fig 9:**
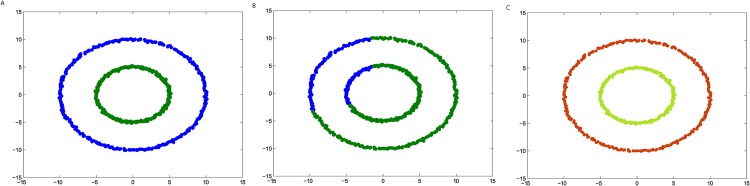
The clustering results of DBSCAN, DP Clustering and DPAM using Donut dataset. (A) DBSCAN (*MinPts* = 1, *Eps* = 2), (B) DP (*percent* = 5), (C) DPAM.

**Table 3:** Comparison of 3 clustering algorithms on synthetic datasets.

Algorithms	Jain		Spiral		Flame		Donut	
	*FM*	*Time*(*s*)	*FM*	*Time*(*s*)	*FM*	*Time*(*s*)	*FM*	*Time*(*s*)
*DBSCAN*	1.0000	0.3460	1.0000	0.1975	0.9659	0.1157	1.0000	0.2498
*DPClustering*	1.0000	0.4820	1.0000	0.4055	1.0000	0.4189	0.5469	0.3998
*DPAM*	1.0000	0.5107	1.0000	0.3191	0.9463	0.2220	1.0000	1.4701

For datasets Jain and Spiral, three algorithms all obtain best results. For datasets Flame, results of DPAM are close to the best. For dataset Dount, DPAM obtains correct results, DP cannot identify it. We take more time than others when algorithms run once, but it may take a long time to learn how to select appropriate parameter values for DBSCAN and DP. Thus DPAM needs less learning time and obtains good results.

### The UCI datasets

**Fig 10:**
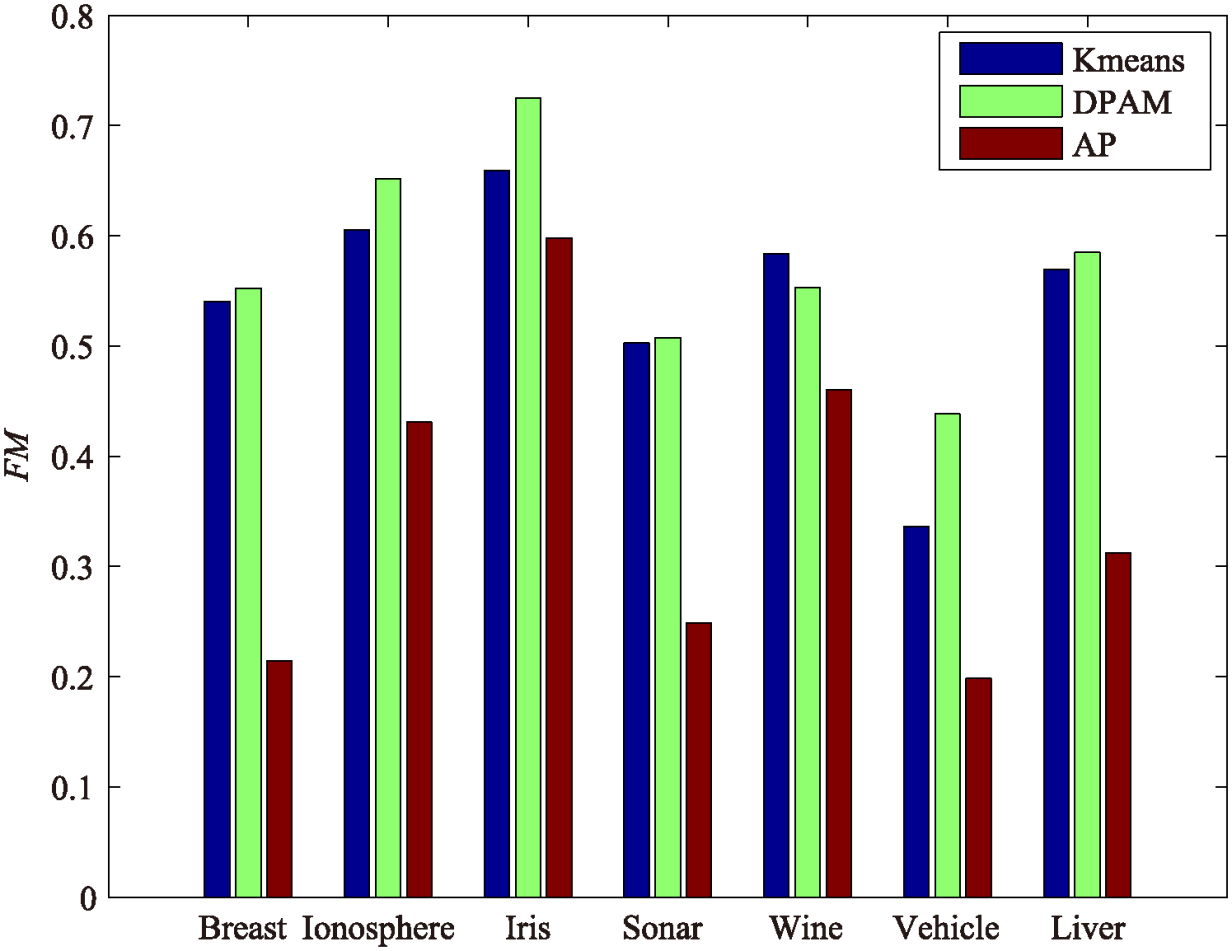
The clustering results of k-means, DPAM and AP with *FM*.

For assessing the robustness of DPAM, we conduct experiments on high-dimensional datasets compared with K-means and affinity propagation (AP) as shown in Fig 10. We set parameter *k* equal to the real class number in K-means. DPAM achieves better performance than k-means and AP. Although the performance of DPAM is just a bit lower than AP in Wine datasets, while Wine is clustered into 3 groups by DPAM, AP groups Wine into 6 clusters. Obviously, DPAM performs better than AP, because the actual number of classes in Wine is 3.

From the above experimental results, one can see that our method has distinct advantage of effectively and straightforwardly dealing with low-dimensional multi-density spatial data and high-dimensional data requiring no parameter adjustment and no human intervention. Users may require no domain knowledge. However, we also point out its current limitation: DPAM may not well recognize the linked points between clusters. For example, DPAM does not work well with the Flame dataset, but DP Clustering can achieve good results on the Flame dataset by adjusting the parameter *d*_*c*_. This means further investigation is needed to improve DPAM and make it applicable to a wider range of density spatial data.

## Conclusions and future work

In this research we present a new approach to adaptively obtain optimal density clustering results. Density propagation based adaptive density clustering adopts a three-stage strategy to clustering low-dimensional density spatial data, and it also perform well on high-dimensional data. We report the promising performance of our approach for clustering different datasets and experimental results indicate that our approach overcomes the limitations of some existing clustering algorithms.

We also point out the limitation of our approach and the potential improvement upon it. When dealing with data with complex structure, such as the linked spatial data, we still need further investigation on how to improve the performance of our proposed density clustering algorithms. Finally, we will also consider applying DPAM to more real-world problems, including material characterization and selection in manufacturing.
